# Genetic effects of *BDKRB2* and *KNG1* on deep venous thrombosis after orthopedic surgery and the potential mediator

**DOI:** 10.1038/s41598-018-34868-9

**Published:** 2018-11-26

**Authors:** Qingfeng Wang, Guoping Cheng, Xiaohui Wang, Dandan Wang, Yanmei Yang, Ke Chen, Jiumin Ye, Zhong Qing

**Affiliations:** 1 Department of Research and Intellectual Property, Orthopedic Hospital of Henan Province, Luoyang, Henan China; 2 Department of clinical laboratory, The First Affiliated Hospital, and college of Clinical Medicine of Henan University of Science and Technology, Luoyang, Henan China; 3 Department of Hand Surgery, Orthopedic Hospital of Henan Province, Luoyang, Henan China; 4 Department of Hip Injury and Disease, Orthopedic Hospital of Henan Province, Luoyang, Henan China; 5 Department of Anesthesiology, Honghui Hospital, Xi’an Jiaotong University, Xi’an, Shaanxi China; 6 Department of Joint Surgery, Honghui Hospital, Xi’an Jiaotong University, Xi’an, Shaanxi China

## Abstract

Deep venous thrombosis (DVT) is a common complication of orthopedic surgery. Genetic risk factors and high heritability carried a substantial risk of DVT. In this study, we aimed to investigate the potential association in the Han Chinese population between the polymorphisms of *BDKRB2* and *KNG1* and DVT after orthopedic surgery (DVTAOS). A total of 3,010 study subjects comprising 892 DVT cases and 2,118 controls were included in the study, and 39 single nucleotide polymorphisms (SNPs) in total (30 for *BDKRB2* and 9 for *KNG1*) were chosen for genotyping. Two SNPs, rs710446 (OR = 1.27, *P* = 0.00016) and rs2069588 (OR = 1.29, *P* = 0.00056), were identified as significantly associated with DVTAOS. After adjusting for BMI, the significance of rs2069588 decreased (*P* = 0.0013). Haplotype analyses showed that an LD block containing rs2069588 significantly correlated with the DVTAOS risk. Moreover, bioinformatics analysis indicated that hsa-miR-758-5p and *BDKRB2* formed miRNA/SNP target duplexes if the rs2069588 allele was in the T form, suggesting that rs2069588 may alter *BDKRB2* expression by affecting hsa-miR-758-5p/single-nucleotide polymorphism target duplexes. Our results demonstrate additional evidence supporting that there is an important role for the *KNG1* and *BDKRB2* genes in the increased susceptibility of DVTAOS.

## Introduction

Deep venous thrombosis (DVT) is a common disorder, in which is a blood clot occurs inside a vein; DVT has several risk factors, including acquired, inherited and mixed. Major surgery and orthopedic surgery, as acquired risk factors, carry a substantial risk of DVT. Clinically, 40% to 60% of patients acquired DVT after orthopedic surgery; 4% to 10% of those patients developed pulmonary embolism (PE), of which 5% die^1^. Therefore, identifying patients with a higher risk of DVT after orthopedic surgery (DVTAOS) is essential in guiding diagnosis and reducing the death rate. A growing body of literature has indicated that all strong, moderate, and weak genetic risk factors with high heritability carry a substantial risk of DVT^2^. Tissue-type plasminogen activator (t-PA) and plasminogen activator inhibitor-1 (PAI-1) are important biochemical constituents of the fibrinolytic system, which affects DVT^3^. This study found evidence that the polymorphisms in the fibrinolytic system might influence PAI-1 and t-PA^3^. With hundreds of mutations responsible for defective genes, deficiencies of antithrombin could lead to a high risk of DVT^2^.

A previous study has shown that Kng1-deficiency is associated with a decrease in thrombosis^4^. With the development of high-throughput DNA sequencing techniques, genome-wide association studies (GWASs) have provided supportive evidence for the polygenic nature of many complex diseases susceptibility^5–11^ and have identified some SNPs that contribute to the risk of DVT, such as Kininogen-1(KNG1)^12^. However, these results cannot explain the genetic portion of DVT. So far, the molecular mechanisms of DVT remain unknown. The *KNG1* gene, which is found on human chromosome 3, also known as alpha-2-thiol proteinase inhibitor, encodes a high-molecular-weight kininogen (HMWK) and low-molecular-weight kininogen (LMWK). KNG1 is a constituent of the blood coagulation system, as is the HMWK protein. KNG1 is associated with the genetic factors of activated partial thromboplastin time (aPTT), which is a risk marker of DVT^13^. In addition, Hu had proven that variants of KNG1 genes potentiate the risk of thrombosis in ischemic stroke in the Chinese population^14^. The association between KNG1 and DVTAOS is largely unknown, so it is urgent to study this mechanism. In addition, the protected mechanism for the thrombosis of Bradykinin receptor B2 knockout (BKB2R^−/−^) in mice via the plasma kallikrein/kinin and renin angiotensin systems also provides new genic factor insights for DVT^15^. The *BDKRB2* gene in humans encodes a G-protein coupled receptor for bradykinin called BKB2R; this receptor elicits many responses, including vasodilation, edema, smooth muscle spasm and pain fiber stimulation. The disfunction of BDKRB2 plays an essential role in some physiological and pathological processes, such as kidney development^16^, the inflammatory process in osteoarthritis^17^ and hepatocellular carcinoma progression^18^. BK, which is antithrombotic for endothelium binding to BKB2R in the intravascular system, promotes blood flow through NO, prostacyclin formation and tPA liberation^19,20^. Interestingly, BDKRB2 knockout mice are protected from thrombosis by increased nitric oxide and prostacyclin^20^. Considering the potential relationship between BDKRB2 and thrombosis, the underlying association and mechanism between BDKRB2 variants and DVT are worth investigation.

Considering the genetic heterogeneity of disease occurrence and the different etiology of DVT, the exploration of possible associations between KNG1/BDKRB2 and DVT among the Han Chinese population may shed light on the underlying mechanisms of DVT. Above all, we reasoned that alleles of KNG1 and BDKRB2 might be associated with an increased risk of DVTAOS. To test the above hypothesis, we aimed to investigate whether common variants in BDKRB2 and KNG1 have interactive effects with DVTAOS. Providing optimal thromboprophylaxis to a patient who is at DVT risk will ensure the best reductions in DVT-related morbidity and mortality from a genetic perspective via this research.

## Methods

### Study subjects

In the current study, we recruited 3,010 subjects undergoing knee or hip orthopedic surgery at Luoyang Orthopedic Hospital of Henan Province (Luoyang, China) from August 2012 to July 2017. Of these, 892 were diagnosed with DVTAOS and were designated as a case group; and 2,118 had no typical DVT symptoms or signs and were designated as a control group. Each subject received anticoagulants routinely within 5 hours of surgery. Two independent sonographers performed preliminary screening using lower-extremity color-Doppler ultrasound, and all subjects were assessed for DVT postoperatively within 6 days. When it was difficult to obtain a definitive diagnosis, venography was used to confirm the diagnosis. Subjects with a history of venous thromboembolism or clinical evidence of venous thromboembolism were excluded from the study. A total number of 211 study subjects were excluded for this criterion. All subjects were unrelated Han Chinese individuals. The clinical data and characteristics of all the subjects were measured or recorded and are summarized in Table 1. Both body mass index (BMI) and hyperlipidemia status showed significant difference between the DVTAOS case group and the controls. Written informed consent was obtained from all subjects. This study was performed in accordance with the ethical guidelines of the Declaration of Helsinki (version 2002) and was approved by the Medical Ethics Committee of Luoyang Orthopedic Hospital of Henan Province.

**Table 1:** Characteristic information for the 3,010 study subjects.

	Case (N = 892)	Controls (N = 2,118)	Statistics	*P*
Age	59.3 ± 6.3	58.9 ± 8.1	*T* = 1.2	0.23
BMI	25.9 ± 1.6	25.5 ± 1.6	*T* = 6.3	4.93 × 10^−10^
Gender (%)				
Male	405 (45)	970 (46)		
Female	487 (55)	1148 (54)	χ^2^ = 0.03	0.87
Site (%)				
hip	519 (58)	1224 (58)		
knee	373 (42)	894 (42)	χ^2^ = 0.03	0.87
Hypertension (%)				
Yes	253 (28)	585 (28)		
No	639 (72)	1533 (72)	χ^2^ = 0.14	0.71
Diabetes (%)				
Yes	43 (5)	95 (4)		
No	849 (95)	2023 (96)	χ^2^ = 0.09	0.76
Hyperlipidemia (%)				
Yes	262 (30)	523 (25)		
No	630 (70)	1595 (75)	χ^2^ = 6.89	0.009
Smoking (%)				
Yes	156 (17)	351 (17)		
No	736 (83)	1767 (83)	χ^2^ = 0.31	0.58
Alcohol (%)				
Yes	128 (14)	281 (13)		
No	764 (86)	1837 (87)	χ^2^ = 0.54	0.46

### SNP selection & Genotyping

Tag SNPs were searched in the study. Tag SNPs of *BDKRB2* and *KNG1* with minor allele frequency (MAF) >= 0.1 based on 1000 genome CHB data were selected for genotyping, and the r^2^ criterion used for tagging was 0.6. In total, 39 SNPs (30 for *BDKRB2* and 9 for *KNG1*) were chosen (Supplemental Table S1). Genomic DNA was isolated from the peripheral blood using a Tiangen DNA extraction kit (Tiangen Biotech Co. Ltd, Beijing, China) according to the manufacturer’s protocol. SNP genotyping was performed using a Sequenom MassARRAY platform with iPLEX GOLD chemistry (Sequenom, San Diego, CA, USA) based on the manufacturer’s protocols. The results were processed using Sequenom Typer 4.0 software^21^, and the genotype data were generated from the samples. Genotyping was conducted by laboratory personnel blinded to case-control status, and the genotyping results, data entry and statistical analyses were independently reviewed by two authors. We randomly reperformed the analysis on 5% of the sample, with a concordance of 100%.

### Statistical analyses

The genetic association between our selected tag SNPs and DVTAOS status were investigated in a three-step procedure. (1) We fit simple logistic models for each SNP and screened out the significant hits; (2) we examined the correlations between these significant SNPs and the clinical variables which were associated with DVTAOS status as shown in Table 1 (BMI and hyperlipidemia status) to obtain the clinical variables which could serve as potential mediators, and (3) we performed mediation analysis to examine the direct effect of targeted SNPs after adjusting those relevant clinical variables. In addition, we also constructed the linkage disequilibrium (LD) blocks for our genetic data and performed haplotype-based association analyses. Bonferroni corrections were applied for multiple comparisons. Plink^22^ was utilized to perform the genetic association analyses. Haploview^23^ was used to make LD plots. Genotypes of SNPs were coded in the additive mode in all statistical analyses.

### Expression quantitative trait loci (eQTL) and bioinformatics analyses

We investigated the eQTL pattern of the significant SNPs using data extracted from the GTEx database^24^. The potential functional significance of the significant SNPs was examined by SIFT (for nonsynonymous SNPs)^25^ and the PolymiRTS Database (for SNPs located at regulatory region)^26^.

## Results

### Significant SNPs identified from simple models

Two SNPs, rs710446 (OR = 1.27, *P* = 0.00016) and rs2069588 (OR = 1.29, *P* = 0.00056), were identified as significantly associated with DVTAOS status through simple logistic models (Table 2). SNP rs710446 was a nonsynonymous change of *KNG1*, which altered the amino acid from IIe to Thr. SNP rs2069588 was located at the 3′ untranslated region (UTR) of the gene *BDKRB2*.

**Table 2:** Genetic associations between single polymorphisms and DVT after orthopedic surgery.

GENE	CHR	SNP	POS	A1	MAF	OR	SE	L95	U95	STAT	*P*
*KNG1*	3	rs710446	186459927	C	0.27	1.27	0.06	1.12	1.43	3.77	0.00016
*BDKRB2*	14	rs2069588	96708667	T	0.17	1.29	0.07	1.12	1.48	3.45	0.00056

CHR: chromosome; POS:position;A1:tested allele; MAF: minor allele frequency; SE:standard error; L95: lower bound of 95% confidence interval; U95: upper bound of 95% confidence interval; STAT: statistics.

Threshold for *P* values used here was 0.05/39≈0.001.

### Mediation analyses for SNP rs2069588

SNP rs2069588 was found to be significantly correlated with BMI (*P* = 0.02, Supplemental Table S2). Combined with the result that BMI was strongly associated with the disease status of DVTAOS (*P* = 4.93 × 10^−10^, Table 1), it might be a potential mediator between rs2069588 and DVTAOS. We performed a mediation analysis to examine the effect of SNP rs2069588 on DVTAOS with or without BMI included as a covariate. After adjusting for BMI, the significance of rs2069588 decreased slightly to *P* = 0.0013 (Table 3). The significance of BMI also decreased from 10^−10^ to the 10^−9^ level, which was obtained in a model with BMI and DVTAOS status only.

**Table 3:** Mediation analysis of rs2069588 and DVT with or without BMI adjusted.

Variables	Model1*				Model2**			
	OR	SE	STAT	*P*	OR	SE	STAT	*P*
rs2069588	1.29	0.07	3.45	5.59 × 10^−4^	1.27	0.07	3.21	0.0013
BMI	—	—	—	—	1.16	0.02	5.96	2.47 × 10^−9^

*Univariate model with genotypes of rs2069588 only.

**Multivariate model including both genotypes of rs2069588 and BMI.

### Haplotype-based association analyses

A total number of 10 2-SNP LD blocks were constructed for both *KNG1* and *BDKRB2* (Supplemental Figure S1 and S2). One LD block, rs2069583-rs2069588 from *BDKRB2*, was significantly associated with DVTAOS status (*P* = 0.0001, Supplemental Table S3). Since rs2069588 was also identified as significant in single marker-based analyses, the significance of the LD block of rs2069583-rs2069588 could be considered a replicate for the results of the single marker-based analyses.

### Significant eQTL and functional significance for rs710446 and rs2069588

**Figure 1:**
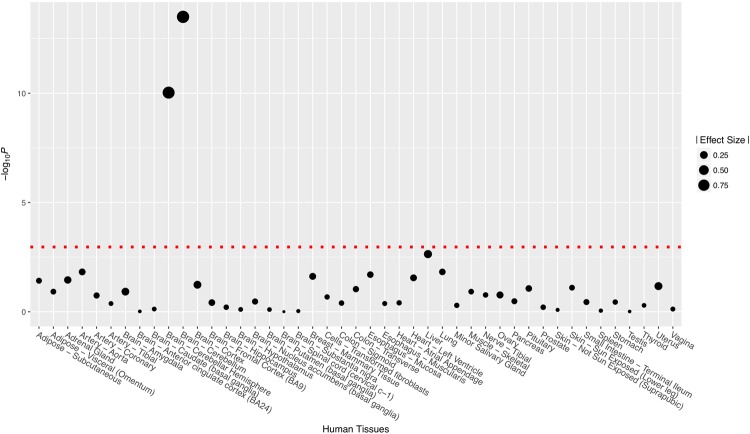
eQTL pattern for rs2069588 on the gene *BDKRB2* based on data extracted from the GTEx database. Threshold of the *P* values was 0.05/45≈0.001.

**Figure 2:**
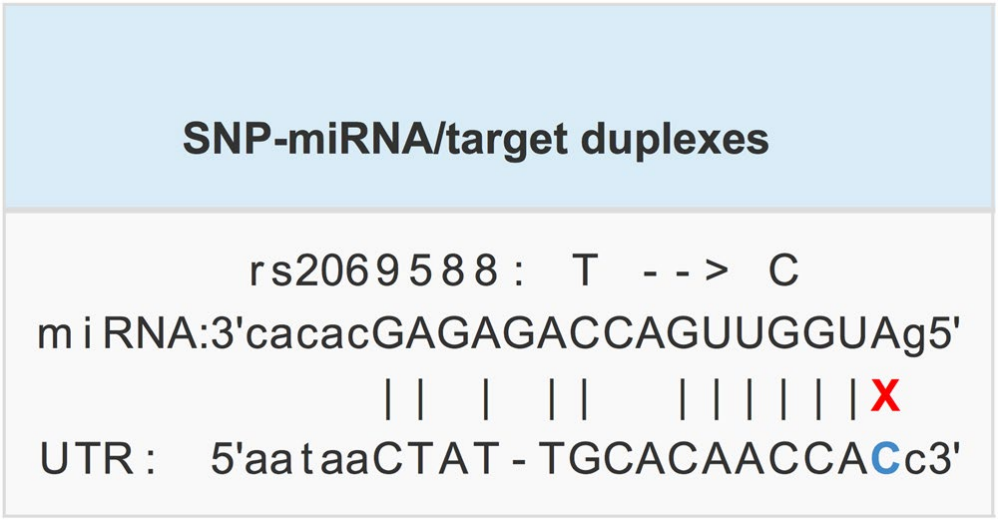
The allele of rs2069588 disrupts miRNA/SNP target duplexes. Hsa-mir-758-5p and *BDKRB2* produce miRNA/SNP target duplexes if the rs3025039 allele is T.

Significant eQTL signals (Fig. 1) were identified for rs2069588 on *BDKRB2* from tissue of the cerebellum (NES = −0.95, *P* = 3.20 × 10^−14^) and cerebellar hemisphere (NES = −0.89, *P* = 9.40 × 10^−11^). No significant eQTL signal was identified for rs710446 on *KNG1*. The full results of eQTL analyses are summarized in Supplemental Table S4 and S5. SIFT predicted that rs710446 had very limited functional consequences on the protein encoded by *KNG1* (rated “tolerated” for all changes), although it altered one amino acid. According to the genomic database from the University of California, Santa Cruz (http://genome.ucsc.edu/), rs2069588 acts as a 3′ untranslated region (3′ UTR) variant, which may affect microRNA binding. We used a free online tool (http://bioinfo.life.hust.edu.cn) to examine the predicted target gain or loss in microRNA binding and found that the C allele of rs2069588 causes a loss of microRNA/SNP target duplexes of hsa-miR-758-5p and *BDKRB2* (Fig. 2). On the other hand, T allele of rs2069588 is a stable microRNA binding site at the 3′ UTR region of *BDKRB2*.

## Discussion

In this study, we investigated the genetic association between DVTAOS and two loci: *KNG1* and *BDKRB2*. Significant evidence for both loci was found to establish their association with DVTAOS. Early studies based on European populations have provided supportive evidence for the association between DVT and rs710446^12^. Our results on rs710446 successfully replicated these previous findings in the Han Chinese population despite focusing on DVTAOS, which is a postoperative complication of DVT. The direction of the effect and the OR in the previous study were similar to ours (OR = 1.19 to OR = 1.27). On the other hand, we identified an SNP, rs2069588, located at the 3′UTR, conferring risk of DVTAOS based on the Han Chinese population. To the best of our knowledge, our study is the first to establish the genetic association between the gene *BDKRB2* and DVTAOS.

In addition to exploring the potential genetic effects in the simple model, we also noticed some potential mediators in the effects between SNPs and DVTAOS. In our study, we statistically proved that BMI might partly mediate the effect of rs2069588 to DVTAOS. From the results of our mediation analyses, we can see that when both BMI and rs2069588 were included in the logistic model, the effect size and significance of rs2069588 were reduced. Combined with the other results, which showed that BMI was significantly associated with the disease status of DVTAOS and genotypes of rs2069588, our findings indicate that part of the effect of rs2069588 on DVTAOS was mediated through BMI. In addition, BMI cannot be a confounder because the direction of effect between BMI and rs2069588 should be from rs2069588 to BMI and was unlikely to be reversed. Interestingly, it seems that our findings on the role of the mediator of BMI were not only based on statistical analyses of our data but could be supported by previous publications. BMI and obesity have long been known as risk factors for DVT^27,28^. On the other hand, although no evidence has been published to establish a direct connection between *BDKRB2* and obesity or BMI, *BDKRB2* was reported to be significantly associated with other human metabolism-related traits, including body fat modulation^29^ and diabetes^30^. In addition, interaction effects have also been identified between the SNPs of *BDKRB2* and physical activity and blood pressure^29^. These previous findings indicated that the effect between rs2069588 and BMI might also be mediated by some other underlying factors in our study. However, the mediation effect of BMI was not identified for rs710446.

Our eQTL analyses based on the data extracted from the GTEx database revealed some significant signals for rs2069588 in *BDKRB2*. However, it is unclear why these eQTL signals were identified in human brain tissues. We know that eQTLs are very common in the human genome and are far more common than disease association signals. A great many SNPs have “significant” eQTL data in GTEx simply because they represent points along an eQTL association peak. Therefore, we need to be careful to interpret the results of eQTL analyses and more experimental studies are still needed in the future.

The SNP rs2069588 of *BDKRB2* was predicted to be a miR-758-5p binding site and its C allele may cause a loss of the original miR-758-5p binding site. We hypothesize that its T allele as a stable miR-758-5p binding site in the 3′ UTR region of *BDKRB2*, and the T allele down regulates the gene expression of *BDKRB2* by binding miR-758-5p compared to the C allele. It is interesting to note that the T allele of rs2069588 is the risk allele for DVTAOS and increased the risk of DVTAOS by approximately 30% in our data. Increasing evidence indicates that bradykinin plays critical roles in coagulation and fibrinolysis^15,31^. Previous studies revealed that by binding to the constitutive bradykinin B2 receptor in the intravascular compartment, bradykinin promotes prostacyclin and plasmin formation and tissue plasminogen activator (t-PA) liberation^20^, which can inhibit thrombosis. Evidence indicated that bradykinin binding to a-thrombin would substantially reduce the fibrinogen to fibrin conversion and inhibit clot formation^32^. Moreover, exogenous bradykinin attenuated deep venous thrombosis via reduced tissue factor (TF) expression at the mRNA and protein level in the mouse model^33^. Hence, the reduction of bradykinin B2 receptor expression may result in upregulation of TF factor expression, and initiate the physiological coagulation cascade, eventually leading to DVT.

A potential limitation of this study is the potential population stratification (PS) which might cause false positive results as a confounder. As ours was a candidate gene-based study, we cannot perform some statistical methods, such as principal component analysis or genomic control, to properly control PS. However, on the other hand, in order to restrict population stratification we have applied some criteria, such as recruiting samples by restricting the subjects with a stable living region^34–36^, during our sample collection process to reduce the genetic heterogeneity of our study subjects^37,38^. Another limitation is that the strategy of screening candidate SNPs was not rigorous enough to discover all potential functional SNPs, including rare variants. It is possible that these variants may contribute to DVTAOS risk in an unknown manner or in linkage disequilibrium with other undetected variants that confer a risk for DVTAOS. In addition, excluding patients with a history or clinical evidence of venous thromboembolism might cause a problem in generalization of the results of this study. Finally, as is known, the onset and development of DVTAOS can involve more genes in a complicated manner, which might be a promising direction for enrolling more related genes in future studies. In particular, future functional experiments would be valuable to understand the roles of hsa-miR-758-5p and the T allele of rs2069588 in DVTAOS. Thus, our results should be considered preliminary, and follow-up studies will be required in the future. More molecular biology experiments and model animal based studies are still needed in future to unravel the underlying biomedical mechanisms of rs710446 and its effect on DVTAOS. The aPTT assay should be performed to examine whether this SNP affects coagulation activity. In addition, further functional studies should be conducted to investigate whether this nonsynonymous SNP is more susceptible to cleavage by activated Factor XIIa or plasma kallikrein.

In sum, in this study, we gained evidence of the genetic association between DVTAOS and two loci: *KNG1* and *BDKRB2* in Han Chinese individuals. Further analyses have revealed the effects of *BDKRB2* on DVT, which might be partly mediated through BMI or some other BMI-related underlying factors. In the future, a comprehensive study of more representative SNPs, different races, larger sample sizes, and functional experiments would be desired to confirm our findings and understand the effects of KNG1 and BDKRB2 on the risk of DVTAOS.

## Electronic supplementary material

Supplemental Materials
