# Supplementary material for: Genetic effects of BDKRB2 and KNG1 on deep venous thrombosis after orthopedic surgery and the potential mediator
Source: Sci Rep. 2018 Nov 26;8:17332. doi: 10.1038/s41598-018-34868-9 (PMC6255904; doi:10.1038/s41598-018-34868-9)
Supplement: Supplementary file 1 — Supplemental Materials [file 41598_2018_34868_MOESM1_ESM.docx]

***Title:*** Genetic effects of *BDKRB2* and *KNG1* on deep venous thrombosis after orthopedic surgery and the potential mediator

***Author names and affiliations***: Qingfeng Wang ^1*^, Guoping Cheng ^2*^, Xiaohui Wang ^3^, Dandan Wang ^1^, Yanmei Yang ^1^, Ke Chen ^4^, Jiumin Ye ^5^ and Zhong Qing ^6^

^1^ Department of Research and Intellectual Property, Orthopedic Hospital of Henan Province, Luoyang, Henan, China;

^2^ Department of clinical laboratory,The First Affiliated Hospital, and college of Clinical Medicine of Henan University of Science and Technology, Luoyang, Henan, China;

^3^ Department of Hand Surgery ,Orthopedic Hospital of Henan Province, Luoyang, Henan, China;

^4^ Department of Hip Injury and Disease ,Orthopedic Hospital of Henan Province, Luoyang, Henan, China;

^5^ Department of Anesthesiology, Honghui Hospital, Xi’an Jiaotong University, Xi'an, Shaanxi, China;

^6^ Department of Joint Surgery, Honghui Hospital, Xi’an Jiaotong University, Xi'an, Shaanxi, China.

* These authors contributed equally to the work.

***Corresponding Author***:

Jiumin Ye, M.D. & Ph.D., Department of Anesthesiology, Honghui Hospital, Xi’an Jiaotong University, No.555, Youyi East Road, Xi'an, Shaanxi, China, 710054.

Tel: 86-29-87894727; Fax: 86-29-87894727; E-mail: anejimye@163.com

Zhong Qing, M.D. & Ph.D., Department of Joint Surgery, Honghui Hospital, Xi’an Jiaotong University, No.555, Youyi East Road, Xi'an, Shaanxi, China, 710054.

Tel: 86-29-62818386; Fax: 86-29-62818386; E-mail: drqizhg@163.com

Supplemental Table S1. Basic information for 39 selected SNPs. from *KNG1* and *BDKRB2*.

| CHR | POS | SNP | ALLELES | FUNC | GENE | MAF | HWE |
| --- | --- | --- | --- | --- | --- | --- | --- |
| 3 | 186437513 | rs13084325 | A/G | intron | *KNG1* | 0.11 | 0.64 |
| 3 | 186443617 | rs266725 | C/G | intron | *KNG1* | 0.33 | 0.80 |
| 3 | 186461216 | rs266760 | C/T | intron | *KNG1* | 0.14 | 0.31 |
| 3 | 186440559 | rs5029999 | C/T | intron | *KNG1* | 0.28 | 0.75 |
| 3 | 186440986 | rs5030002 | A/G | intron | *KNG1* | 0.49 | 0.46 |
| 3 | 186452415 | rs5030058 | G/T | intron | *KNG1* | 0.32 | 0.96 |
| 3 | 186460877 | rs5030091 | C/T | intron | *KNG1* | 0.41 | 0.42 |
| 3 | 186461349 | rs5030095 | C/G | intron | *KNG1* | 0.26 | 0.50 |
| 3 | 186459927 | rs710446 | A/G | missense | *KNG1* | 0.27 | 0.16 |
| 14 | 96673094 | rs11160321 | C/G | intron | *BDKRB2* | 0.17 | 0.35 |
| 14 | 96689875 | rs11628424 | G/T | intron | *BDKRB2* | 0.11 | 0.64 |
| 14 | 96671745 | rs117840361 | C/T | intron | *BDKRB2* | 0.16 | 0.75 |
| 14 | 96686296 | rs11847625 | A/C | intron | *BDKRB2* | 0.22 | 0.53 |
| 14 | 96675022 | rs12433275 | C/T | intron | *BDKRB2* | 0.17 | 1.00 |
| 14 | 96671139 | rs1799722 | C/T | near-gene-5 | *BDKRB2* | 0.47 | 0.30 |
| 14 | 96673409 | rs1959053 | C/T | intron | *BDKRB2* | 0.25 | 0.77 |
| 14 | 96702587 | rs2069575 | A/G | intron | *BDKRB2* | 0.19 | 0.95 |
| 14 | 96703795 | rs2069578 | A/G | intron | *BDKRB2* | 0.37 | 0.49 |
| 14 | 96705694 | rs2069583 | A/G | intron | *BDKRB2* | 0.36 | 0.64 |
| 14 | 96708667 | rs2069588 | C/T | untranslated-3 | *BDKRB2* | 0.17 | 0.17 |
| 14 | 96708782 | rs2069590 | A/T | untranslated-3 | *BDKRB2* | 0.45 | 0.43 |
| 14 | 96680903 | rs4900315 | C/T | intron | *BDKRB2* | 0.49 | 0.73 |
| 14 | 96679395 | rs4905461 | C/G | intron | *BDKRB2* | 0.35 | 0.34 |
| 14 | 96697364 | rs4905474 | A/G | intron | *BDKRB2* | 0.39 | 0.41 |
| 14 | 96676356 | rs58344703 | A/C | intron | *BDKRB2* | 0.21 | 1.00 |
| 14 | 96673398 | rs60546995 | C/T | intron | *BDKRB2* | 0.21 | 0.52 |
| 14 | 96690826 | rs61193624 | A/G | intron | *BDKRB2* | 0.25 | 0.95 |
| 14 | 96690488 | rs7150828 | A/G | intron | *BDKRB2* | 0.47 | 0.46 |
| 14 | 96693967 | rs7154191 | C/T | intron | *BDKRB2* | 0.11 | 0.38 |
| 14 | 96682353 | rs7155797 | C/T | intron | *BDKRB2* | 0.46 | 0.51 |
| 14 | 96686622 | rs73359933 | A/G | intron | *BDKRB2* | 0.17 | 0.49 |
| 14 | 96699456 | rs75139981 | C/T | intron | *BDKRB2* | 0.13 | 0.85 |
| 14 | 96700297 | rs76128588 | A/G | intron | *BDKRB2* | 0.14 | 0.30 |
| 14 | 96705409 | rs76228306 | C/G | intron | *BDKRB2* | 0.11 | 1.00 |
| 14 | 96683768 | rs79521094 | C/T | intron | *BDKRB2* | 0.11 | 0.64 |
| 14 | 96684156 | rs80026658 | C/T | intron | *BDKRB2* | 0.17 | 1.00 |
| 14 | 96688767 | rs8012552 | C/T | intron | *BDKRB2* | 0.47 | 0.46 |
| 14 | 96675933 | rs8016905 | A/C | intron | *BDKRB2* | 0.34 | 0.77 |
| 14 | 96684198 | rs945039 | A/C | intron | *BDKRB2* | 0.43 | 0.56 |

CHR: chromosome; POS: position; FUNC: functional region; MAF:minor allele frequency; HWE: *P* values of Hardy-Weinberg Equilibrium tests.

Supplemental Table S2. Correlations between SNP rs710446/rs2069588 and relevant clinical variables.

|  | BMI | | | Hyperlipidemia | | |
| --- | --- | --- | --- | --- | --- | --- |
| SNP | Beta | STAT | *P* | OR | STAT | *P* |
| rs710446 | -0.0009 | -0.02 | 0.99 | 1.01 | 0.01 | 0.93 |
| rs2069588 | 0.13 | 2.33 | 0.02 | 1.15 | 3.25 | 0.07 |

For BMI, we fitted a linear regression to evaluate its potential correlation with SNPs. For hyperlipidemia, we performed a logistic regression.

Supplemental Table S3. Results of haplotype association analyses.

| LOCUS | HAPLOTYPE | F_A | F_U | χ^2^ | DF | *P* | SNPs |
| --- | --- | --- | --- | --- | --- | --- | --- |
| *KNG1* | OMNIBUS | NA | NA | 0.285 | 2 | 0.8670 | rs5029999\|rs5030002 |
|  | TA | 0.28 | 0.28 | 0.129 | 1 | 0.7194 | rs5029999\|rs5030002 |
|  | CA | 0.22 | 0.21 | 0.066 | 1 | 0.7975 | rs5029999\|rs5030002 |
|  | CG | 0.50 | 0.51 | 0.285 | 1 | 0.5937 | rs5029999\|rs5030002 |
| *KNG1* | OMNIBUS | NA | NA | 0.032 | 2 | 0.9840 | rs5030091\|rs266760 |
|  | CA | 0.13 | 0.13 | 0.019 | 1 | 0.8918 | rs5030091\|rs266760 |
|  | CG | 0.28 | 0.28 | 0.022 | 1 | 0.8829 | rs5030091\|rs266760 |
|  | TG | 0.59 | 0.59 | 0.002 | 1 | 0.9675 | rs5030091\|rs266760 |
| *BDKRB2* | OMNIBUS | NA | NA | 0.727 | 2 | 0.6952 | rs11160321\|rs60546995 |
|  | TT | 0.16 | 0.17 | 0.395 | 1 | 0.5296 | rs11160321\|rs60546995 |
|  | CT | 0.05 | 0.05 | 0.406 | 1 | 0.5242 | rs11160321\|rs60546995 |
|  | CC | 0.79 | 0.79 | 0.053 | 1 | 0.8177 | rs11160321\|rs60546995 |
| *BDKRB2* | OMNIBUS | NA | NA | 0.576 | 2 | 0.7497 | rs1959053\|rs12433275 |
|  | AT | 0.16 | 0.17 | 0.348 | 1 | 0.5554 | rs1959053\|rs12433275 |
|  | AC | 0.09 | 0.08 | 0.305 | 1 | 0.5809 | rs1959053\|rs12433275 |
|  | GC | 0.76 | 0.75 | 0.023 | 1 | 0.8783 | rs1959053\|rs12433275 |
| *BDKRB2* | OMNIBUS | NA | NA | 1.037 | 2 | 0.5953 | rs945039\|rs11847625 |
|  | AC | 0.22 | 0.22 | 0.234 | 1 | 0.6288 | rs945039\|rs11847625 |
|  | AG | 0.21 | 0.20 | 0.988 | 1 | 0.3202 | rs945039\|rs11847625 |
|  | GG | 0.57 | 0.58 | 0.164 | 1 | 0.6852 | rs945039\|rs11847625 |
| *BDKRB2* | OMNIBUS | NA | NA | 0.087 | 2 | 0.9573 | rs73359933\|rs8012552 |
|  | GC | 0.17 | 0.17 | 0.007 | 1 | 0.9310 | rs73359933\|rs8012552 |
|  | AC | 0.31 | 0.31 | 0.059 | 1 | 0.8075 | rs73359933\|rs8012552 |
|  | AT | 0.52 | 0.53 | 0.084 | 1 | 0.7719 | rs73359933\|rs8012552 |
| *BDKRB2* | OMNIBUS | NA | NA | 0.151 | 2 | 0.9272 | rs7150828\|rs61193624 |
|  | GT | 0.25 | 0.25 | 0.000 | 1 | 0.9842 | rs7150828\|rs61193624 |
|  | GG | 0.23 | 0.22 | 0.132 | 1 | 0.7164 | rs7150828\|rs61193624 |
|  | AG | 0.52 | 0.53 | 0.103 | 1 | 0.7486 | rs7150828\|rs61193624 |
| *BDKRB2* | OMNIBUS | NA | NA | 1.245 | 2 | 0.5367 | rs4905474\|rs75139981 |
|  | AT | 0.12 | 0.13 | 0.576 | 1 | 0.4479 | rs4905474\|rs75139981 |
|  | AC | 0.28 | 0.26 | 0.942 | 1 | 0.3317 | rs4905474\|rs75139981 |
|  | GC | 0.60 | 0.61 | 0.130 | 1 | 0.7182 | rs4905474\|rs75139981 |
| *BDKRB2* | OMNIBUS | NA | NA | 1.283 | 2 | 0.5266 | rs2069575\|rs2069578 |
|  | AG | 0.19 | 0.19 | 0.316 | 1 | 0.5738 | rs2069575\|rs2069578 |
|  | GG | 0.19 | 0.18 | 1.180 | 1 | 0.2775 | rs2069575\|rs2069578 |
|  | GA | 0.62 | 0.63 | 0.168 | 1 | 0.6819 | rs2069575\|rs2069578 |
| *BDKRB2* | OMNIBUS | NA | NA | 18.180 | 2 | ***0.0001*** | rs2069583\|rs2069588 |
|  | TT | 0.20 | 0.16 | 10.810 | 1 | ***0.0010*** | rs2069583\|rs2069588 |
|  | TC | 0.16 | 0.20 | 11.320 | 1 | ***0.0008*** | rs2069583\|rs2069588 |
|  | AC | 0.64 | 0.64 | 0.024 | 1 | 0.8779 | rs2069583\|rs2069588 |

F_A: haplotype frequency in cases; F_U: haplotype frequency in controls; DF: degree of freedom.

Supplemental Table S4. eQTL results for rs710446 on *KNG1*.

| Gene | SNP | *P* | NES | T-statistic | Tissue |
| --- | --- | --- | --- | --- | --- |
| *KNG1* | rs710446 | 0.0024 | 0.14 | 3.10 | Colon - Transverse |
| *KNG1* | rs710446 | 0.01 | 0.19 | 2.70 | Heart - Atrial Appendage |
| *KNG1* | rs710446 | 0.03 | 0.19 | 2.20 | Small Intestine - Terminal Ileum |
| *KNG1* | rs710446 | 0.08 | 0.13 | 1.80 | Heart - Left Ventricle |
| *KNG1* | rs710446 | 0.22 | 0.14 | 1.20 | Brain - Frontal Cortex (BA9) |
| *KNG1* | rs710446 | 0.25 | 0.14 | 1.20 | Ovary |
| *KNG1* | rs710446 | 0.31 | 0.11 | 1.00 | Brain - Cortex |
| *KNG1* | rs710446 | 0.46 | -0.11 | -0.74 | Cells - EBV-transformed lymphocytes |
| *KNG1* | rs710446 | 0.56 | 0.06 | 0.58 | Testis |
| *KNG1* | rs710446 | 0.62 | 0.02 | 0.50 | Liver |
| *KNG1* | rs710446 | 0.63 | 0.05 | 0.48 | Brain - Anterior cingulate cortex (BA24) |
| *KNG1* | rs710446 | 0.69 | 0.02 | 0.39 | Whole Blood |
| *KNG1* | rs710446 | 0.91 | 0.02 | 0.11 | Uterus |
| *KNG1* | rs710446 | 1.00 | -0.0003 | 0.00 | Lung |

NES: normalized effect size.

Supplemental Table S5. eQTL results for rs2069588 on *BDKRB2*

| Gene | SNP | *P* | NES | T-statistic | Tissue |
| --- | --- | --- | --- | --- | --- |
| *BDKRB2* | rs2069588 | 3.20×10^-14^ | -0.95 | -8.70 | Brain - Cerebellum |
| *BDKRB2* | rs2069588 | 9.40×10^-11^ | -0.89 | -7.20 | Brain - Cerebellar Hemisphere |
| *BDKRB2* | rs2069588 | 0.17 | -0.18 | -1.40 | Ovary |
| *BDKRB2* | rs2069588 | 0.02 | -0.14 | -2.30 | Esophagus - Mucosa |
| *BDKRB2* | rs2069588 | 0.09 | -0.14 | -1.70 | Pituitary |
| *BDKRB2* | rs2069588 | 0.34 | -0.11 | -0.95 | Brain - Nucleus accumbens (basal ganglia) |
| *BDKRB2* | rs2069588 | 0.36 | -0.10 | -0.91 | Small Intestine - Terminal Ileum |
| *BDKRB2* | rs2069588 | 0.62 | -0.07 | -0.50 | Prostate |
| *BDKRB2* | rs2069588 | 0.40 | -0.07 | -0.85 | Colon - Sigmoid |
| *BDKRB2* | rs2069588 | 0.62 | -0.07 | -0.50 | Brain - Hippocampus |
| *BDKRB2* | rs2069588 | 0.21 | -0.06 | -1.20 | Cells - Transformed fibroblasts |
| *BDKRB2* | rs2069588 | 0.39 | -0.06 | -0.87 | Heart - Atrial Appendage |
| *BDKRB2* | rs2069588 | 0.51 | -0.06 | -0.66 | Minor Salivary Gland |
| *BDKRB2* | rs2069588 | 0.79 | -0.03 | -0.26 | Brain - Putamen (basal ganglia) |
| *BDKRB2* | rs2069588 | 0.93 | -0.01 | -0.09 | Brain - Substantia nigra |
| *BDKRB2* | rs2069588 | 0.96 | -0.01 | -0.05 | Brain - Anterior cingulate cortex (BA24) |
| *BDKRB2* | rs2069588 | 0.97 | 0.00 | -0.04 | Testis |
| *BDKRB2* | rs2069588 | 0.99 | 0.00 | 0.01 | Brain - Spinal cord (cervical c-1) |
| *BDKRB2* | rs2069588 | 0.82 | 0.01 | 0.23 | Skin - Not Sun Exposed (Suprapubic) |
| *BDKRB2* | rs2069588 | 0.89 | 0.02 | 0.14 | Spleen |
| *BDKRB2* | rs2069588 | 0.51 | 0.03 | 0.67 | Thyroid |
| *BDKRB2* | rs2069588 | 0.75 | 0.04 | 0.33 | Brain - Caudate (basal ganglia) |
| *BDKRB2* | rs2069588 | 0.78 | 0.04 | 0.28 | Brain - Hypothalamus |
| *BDKRB2* | rs2069588 | 0.42 | 0.04 | 0.81 | Artery - Tibial |
| *BDKRB2* | rs2069588 | 0.75 | 0.04 | 0.32 | Vagina |
| *BDKRB2* | rs2069588 | 0.42 | 0.05 | 0.80 | Esophagus - Muscularis |
| *BDKRB2* | rs2069588 | 0.36 | 0.07 | 0.92 | Stomach |
| *BDKRB2* | rs2069588 | 0.17 | 0.07 | 1.40 | Nerve - Tibial |
| *BDKRB2* | rs2069588 | 0.12 | 0.08 | 1.60 | Muscle - Skeletal |
| *BDKRB2* | rs2069588 | 0.12 | 0.09 | 1.60 | Adipose - Visceral (Omentum) |
| *BDKRB2* | rs2069588 | 0.08 | 0.09 | 1.80 | Skin - Sun Exposed (Lower leg) |
| *BDKRB2* | rs2069588 | 0.04 | 0.10 | 2.10 | Adipose - Subcutaneous |
| *BDKRB2* | rs2069588 | 0.33 | 0.10 | 0.97 | Pancreas |
| *BDKRB2* | rs2069588 | 0.18 | 0.11 | 1.30 | Artery - Coronary |
| *BDKRB2* | rs2069588 | 0.09 | 0.12 | 1.70 | Colon - Transverse |
| *BDKRB2* | rs2069588 | 0.02 | 0.14 | 2.50 | Lung |
| *BDKRB2* | rs2069588 | 0.38 | 0.15 | 0.89 | Brain - Frontal Cortex (BA9) |
| *BDKRB2* | rs2069588 | 0.02 | 0.16 | 2.50 | Artery - Aorta |
| *BDKRB2* | rs2069588 | 0.03 | 0.16 | 2.20 | Heart - Left Ventricle |
| *BDKRB2* | rs2069588 | 0.02 | 0.17 | 2.30 | Breast - Mammary Tissue |
| *BDKRB2* | rs2069588 | 0.04 | 0.21 | 2.10 | Adrenal Gland |
| *BDKRB2* | rs2069588 | 0.12 | 0.25 | 1.60 | Brain - Amygdala |
| *BDKRB2* | rs2069588 | 0.06 | 0.26 | 1.90 | Brain - Cortex |
| *BDKRB2* | rs2069588 | 0.07 | 0.27 | 1.90 | Uterus |
| *BDKRB2* | rs2069588 | 0.002 | 0.29 | 3.10 | Liver |

NES: normalized effect size.


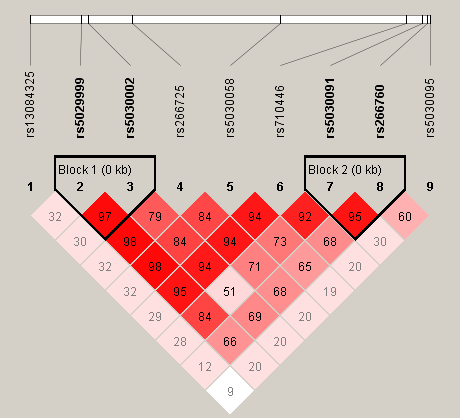


Supplemental Figure S1. LD structure of *KNG1*.


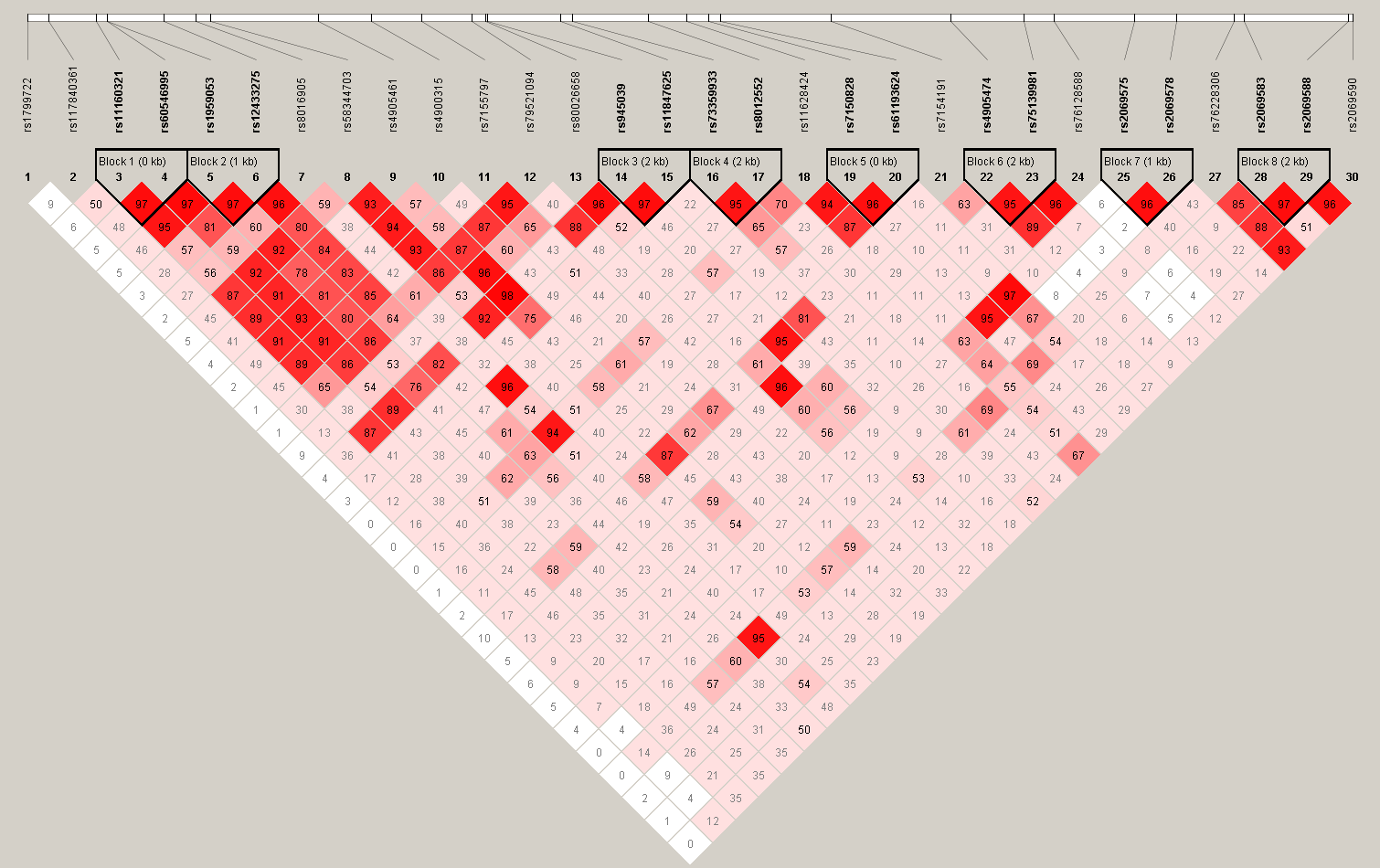


Supplemental Figure S2. LD structure of *BDKRB2.*
